# The predictive value of serial serum estradiol and serial endometrial volume on endometrial receptivity on assisted reproductive technology cycles

**DOI:** 10.1186/s12884-021-03672-1

**Published:** 2021-03-05

**Authors:** R. Silva Martins, A. Helio Oliani, D. Vaz Oliani, J. Martinez de Oliveira

**Affiliations:** 1 Centro Hospitalar Universitário Cova da Beira EPE, Quinta do Alvito, 6200 503 Covilha, Portugal; 2 Centro Investigação Ciências da Saúde – Faculdade Ciências da Saúde, Universidade da Beira Interior, Alameda Infante D, Henrique, 6200 506 Covilha, Portugal

**Keywords:** Endometrial receptivity, Serum estradiol, Endometrial volume, Adjusted endometrial volume, Embryo implantation

## Abstract

**Background:**

Diagnosis of endometrial receptivity is still unclear and conflicting. Despite advances in embryo development during assisted reproductive technologies (ART) cycles, the intricate process of implantation is still matter for debate and research.

**Materials and methods:**

Prospective case control of 169 subjects during ovarian controlled stimulation for ART. Endometrial receptivity assessment to predict clinical pregnancy with serial continuous biochemical (serum estradiol) and biophysical (endometrial volume and adjusted endometrial volume) parameters were used. Both parameters were compared between negative and positive outcome in terms of clinical pregnancy.

**Results:**

No statistical difference was noted between the two groups in terms of demographics and ART procedures and scores. Serum estradiol was significantly higher in the positive group from day 8 after ovarian controlled stimulation. Endometrial volume and adjusted endometrial volume were significantly higher in the positive group as soon as day 6 of ovarian controlled stimulation.

**Conclusions:**

Continuous serum estradiol and 3D endometrial volume and adjusted endometrial volumes may reflect endometrial changes during ART procedures and provide a useful real time tool for clinicians in predicting endometrial receptivity.

## Main text

### Introduction

Since the introduction of assisted conception, many techniques have been developed to improve ovarian stimulation, oocyte retrieval, and embryo culture, but despite this, more than 70% of apparently normal embryos transferred fail to implant. Endometrial receptivity is still a controversial topic and the mechanism to which leads the normal endometrium to become receptive to a healthy embryo unclear. Endometrial differentiation, embryo development and foremost embryo-endometrium interaction leading to implantation requires synchronous and continuous dialogue between these two important components. The physiological and biochemical steps which will allow endometrium to become receptive remains unclear, poorly understood and matter for many discussion and study.

The impact of serum estradiol levels in assisted reproductive techniques (ART), has been debated for over 25 years with conflicting results about the effect of supraphysiological levels of estradiol during controlled ovarian stimulation. Some studies showed a negative impact, while others showed a positive impact in ART outcome. The majority showed no impact [1–5]. High levels of estradiol and its influence on embryonic implantation, and embryonic development is still controversial and unclear. Many has been published regarding the deleterious effects of supraphysiological levels of estradiol on ovarian stimulation for ART procedures. Also, questions have been raised concerning high levels of estradiol and the impairment caused by it on endometrial receptivity. Ovarian hyperstimulation syndrome (OHSS) is an iatrogenic complication of assisted reproduction technology. Estrogen, estradiol, prolactin, histamine and prostaglandins have all been implicated in OHSS.

A metanalysis was conducted in 2019 and no quality evidence was found to support or refute the value of estradiol levels on the day of hCG administration, as a predictor of pregnancy in ART cycles [6]. Conflicting results may relate to the difference between the way the trials were conducted, the difference of stimulation protocol, number of embryos transferred, and the definition of outcome in terms of pregnancy rates.

According to Paulson (2011) the supraphysiological elevation of serum estradiol compromises endometrial receptivity [7]. This elevation plays a definite role in embryo implantation which is claimed to be dose dependent [8]. As a result of multiple follicle maturation, the rise of serum estradiol to supraphysiological levels, alters endometrial receptivity by morphological and biochemical changes produced against this tissue (Simon et al.) [9, 10]. Mirkin et al., 2005 reports that elevated serum estradiol levels have a negative impact on endometrial receptivity especially in fresh embryo transfer cycles [11].

Still clinical trials, contrast with these claims with conflicting results. By Sharara & McClamrock 1999, no significant impact on implantation rates were reported due to the supraphysiological levels of estradiol [12].

In a similar way, ultrasonographic parameters have been attempted to understand endometrial receptivity. Still the results of all these features remain uncertain and also conflicting. Ultrasound can assess endometrium changes during a stimulated ART cycle, in a non-invasive manner. Monitoring both follicle development and endometrium during an ART cycle is normal clinical procedure. Many published studies have conflicting results on this subject but the common feature in all, is the lack of continuity on the endometrial assessment [13–16].

The primary objective of this study is to assess both parameters (endometrial volume and serum estradiol), not only in a single scope pre-determined moment but with a serial prospective continuous evaluation. Endometrium is a responsive tissue that has to undergo serial transformations as a result of the ovarian stimulation. The main goal is to determine the changes and follow up the way both serum estradiol and ultrasound parameters influence this process.

## Material and methods

Prospective case control study of 169 women in ART cycles of diagnosed infertile couples on ART treatment at our institution during a 2-year period (from January 2017 to December 2018). Subjects with canceled treatments prior to oocyte pickup, or with donate gametes, and cryopreserved oocytes were excluded from this study. Also cycles with erroneous or missing date were excluded. Written informed consent was obtained according to the Ethics Committee of our Institution. Inclusion criteria included subjects that had morphologically two good grade cleaved (day 3 of embryo development) embryos for transfer. Only Grade A and B cleaved embryos were included in this selection according to the morphocynetic analysis using ASEBIR embryo assessment criteria. All subjects were submitted to short protocol regimen with antagonists for ovarian controlled stimulation with gonadotropins and were given recombinant human chorionic gonadotropin hormone (rhCG) for induction of ovulation 36 h prior to oocyte pick up.

Demographics and ART parameters were collected for all patients.

Continuous serial serum estradiol levels obtained and ultrasound analysis were performed using the same protocol for all participating subjects, from basal moment (prior to ovarian controlled stimulation) and throughout ovarian controlled stimulation.

Serum levels of estradiol were measured by chemiluminescent enzyme immunoassays (IMMULITE; Diagnostic Products, Los Angeles, CA, USA). Inter-assay and intra-assay coefficients of variation were 9.8 and 9.4%.

Women were scanned by 3D ultrasound using a transvaginal 7.5 MHz transducer (General Electric – GE Volusson E6, USA TM) by a single operator. Sagital midline 2D longitudinal cross section of the uterus was obtained and a volume box was superimposed on the scan image. The volume was captured through the automatic sweep of the transducer. Endometrial volume calculation by 3D ultrasound presented as voxels and geometric information of surfaces in a 3D dataset. The results obtained are then converted to millilitres.

Adjusted Endometrial volume was also obtained as a ratio between endometrial volume calculated on 3D analysis and uterine volume based on 3D volumetric acquisitions which then generated an estimated uterine volume (also in millilitres).

Positive outcome was considered for subjects with positive pregnancy test 12 days after successful embryo transfer and consequently ultrasonographic confirmation of pregnancy with the presence of gestational sac with at least one embryo with positive fetal heart activity.

Data was analysed in Excel 2019 (Microsoft Corp, Redmond, WA) and IBM SPSS statistics v25 (IBM Corp. Armonk, NY).

Continuous variables were analysed with Levene’s test (equality of variances) and visual assessment of the histogram (normality). Results were expressed as mean ± SD, frequency, and percentages. For statistical differences between the means of two groups, t-student for independent samples was applied. Categorical characteristics of patients were compared with χ2 test.

Value of *p* < .05 was considered statistically significant.

Receiver operating characteristic (ROC) curve analysis and comparison of area under curves (AUC) were performed to determine cut-off values of Estradiol, Endometrial Volume and Adjusted Endometrial Volume for the prediction of positive outcome. Investigation of the predictive capability of each logistic model by means of the area under the ROC curve. This curve measures the accuracy of biomarkers to predict positive outcome in endometrial receptivity displayed by the relationship between sensitivity (true positive rate, y-axis) and 1-specificity (false positive rate, x-axis) across the possible threshold values considered. Values of AUC equal to 1 indicate a perfect test, values of AUC higher than 0.9 demonstrate high accuracy and values between 0.7 and 0.9 reflect moderate accuracy of the test.

The authors do not report any conflict of interest.

The study protocol was approved by the Ethics Committee of our Institution (CHCB 22/2017), in accordance with the relevant guidelines and regulations. The study was developed under the research practices described in International Conference Harmonisation (ICH) guidelines, Good Clinical Practices (GCP) and the Declaration of Helsinki.

This study integrates part of a major protocol study and methodological used shares similitude to previous published article by Silva Martins, R [17].

## Results

Subjects were divided into two groups depending on the value of hCG at Day 12 after embryo transfer and ultrasonographic confirmation of clinical pregnancy: 123 on the negative group (72.8%) and 46 on the positive group (27.2%). Demographics characteristics and ART parameters of the 169 subjects are shown in Table 1 and no statistical difference between the two set groups in terms of demographics and ART parameters was met, especially in terms of overall median number of harvested oocytes per cycle defined as the total number of oocytes harvested during oocyte pick up procedure, and rate of collected metaphase II (MII) oocytes. Also, the mean number of cleaved embryos at day 3 of embryo development, and mean number of blastocysts for cryopreservation showed no significant statistical difference between the two set groups.

**Table 1:** Demographics and ART parameters between two Groups

	Negative Group *N* = 123 (72.8%)	Positive Group *N* = 46 (27.2%)	t-Test *p* value
Female Age (in years)	34.94 ± 4.03 (19–39)	34.28 ± 3.35 (25–39)	0.290
Male Age (in years)	36.14 ± 4.76 (22–46)	37.19 ± 5.91 (29–62)	0.832
Time of Infertility (in months)	54.46 ± 33.82 (12–204)	60.22 ± 38.49 (14–192)	0.375
Type of Infertility:			
• Primary	95/123 (77.2%)	38/46 (82.6%)	0.297
• Secondary	28/123 (22.8%)	8/46 (17.4%)	
Male Associated Infertility	28.2%	31.1%	0.654
Body Mass Index (Kg/cm2)	25.8 ± 3.2	26.1 ± 3.4	0.765
Antimullerian hormone (pg/mL)	2.45 ± 2.45 (0.09–16.65)	2.62 ± 2.46 (0.04–13.56)	0.679
Antral follicle count	8.43 ± 5.07 (2–40)	8.63 ± 3.74 (2–20)	0.801
Total dose of gonadotropins (in International Units)	2500.81 ± 812.19 (300–4500)	2508.15 ± 757.91 (450–4500)	0.956
Progesterone levels at Trigger day (ng/mL)	0.88 ± 0.44 (0.01–2.20)	0.78 ± 0.47 (0.01–2.10)	0.188
Number of collected Oocytes	8.25 ± 5.14 (2–22)	10.50 ± 5.20 (2–23)	0.140
Metaphase II Oocytes	6.57 ± 4.22 (2–17)	7.06 ± 4.77 (2–21)	0.150
Number of day 3 embryos	3.18 ± 2.40 (2–12)	3.84 ± 2.65 (2–12)	0.120
Number of blastocysts for vitrification	0.65 ± 1.51 (0–6)	0.86 ± 1.71 (0–9)	0.200

(Positive Group, *N* = 46 and Negative Group, *N* = 123)

Descriptive statistics between two Groups. Mean values with standard deviation (SD)

Serum estradiol levels on basal (prior to ovarian controlled stimulation) was similar between the two groups. Significantly higher levels were noted on the positive outcome group, but it only met statistical significance at Day 8 after ovarian controlled stimulation. (Table 2).

**Table 2:** Serum estradiol measurements between two Groups (in picograms per mililiter – pg/mL)

	Serum Estradiol Levels (pg/mL) Negative Group *N* = 123 (72.8%)	Serum Estradiol Levels (pg/mL) Positive Group *N* = 46 (27.2%)	t-Test p Value
Basal	10.47 ± 2.89	10.39 ± 2.87	0.727
Day 6 after Controlled Ovarian Stimulation	305.10 ± 130.49	381.65 ± 122.46	0.055
Day 8 after Controlled Ovarian Stimulation	840.68 ± 230.49	896.32 ± 222.46	**0.034**
Day 10 after Controlled Ovarian Stimulation	1652.56 ± 607.90	1848.65 ± 599.42	**0.01**
Trigger Day with rhCG	1746.76 ± 654.34	2214.65 ± 612.34	**0.01**

(Positive Group, *N* = 46 and Negative Group, *N* = 123)

Mean values with standard deviation (SD)

**Fig. 1:**
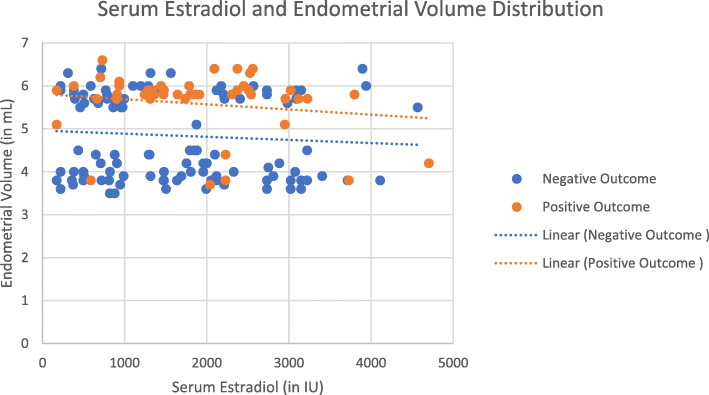
Scatter-plot chart for Serum Estradiol levels and Endometrial volume distribution on trigger day with hCG are displayed. (in blue the negative outcome and in orange the positive outcomes)

Endometrial Volume and Adjusted Endometrial Volume showed statistical difference from Day 6 after Ovarian controlled stimulation (Table 3). Consistently higher values were seen for both of these biophysical markers on the positive group. (Fig. 1).

**Table 3:** Ultrasound parameters between two groups – Endometrial thickness, volume and adjusted endometrial volume at baseline, at day 6, 8 and 10 after controlled ovarian stimulation, at trigger day and at embryo transfer day

		Negative Group *N* = 123 (72.8%)	Positive Group *N* = 46 (27.2%)	t-Test p Value
Basal	Endometrial Thickness (mm)	4.32 ± 0.72	4.22 ± 0.51	0.387
	Endometrial Volume (mm^3^)	2.52 ± 0.71	2.77 ± 0.63	0.54
	Adjusted Endometrial Volume	4.60 ± 1.42	5.51 ± 1.28	0.21
Day 6 after Controlled Ovarian Stimulation	Endometrial Thickness (mm)	6.32 ± 0.96	6.28 ± 0.75	0.827
	Endometrial Volume (mm^3^)	3.08 ± 0.66	3.33 ± 0.57	**0.024**
	Adjusted Endometrial Volume	5.63 ± 1.50	6.67 ± 1.38	**0.001**
Day 8 after Controlled Ovarian Stimulation	Endometrial Thickness (mm)	7.47 ± 0.80	7.96 ± 0.79	**0.01**
	Endometrial Volume (mm^3^)	3.90 ± 0.94	4.40 ± 0.71	**0.002**
	Adjusted Endometrial Volume	7.28 ± 2.67	8.98 ± 2.47	**0.001**
Day 10 after Controlled Ovarian Stimulation	Endometrial Thickness (mm)	8.01 ± 1.04	8.61 ± 0.98	**0.01**
	Endometrial Volume (mm^3^)	4.12 ± 1.01	4.91 ± 0.82	**0.001**
	Adjusted Endometrial Volume	7.60 ± 2.54	9.99 ± 2.61	**0.001**
Trigger Day with rhCG	Endometrial Thickness (mm)	8.53 ± 1.32	9.59 ± 1.44	**0.001**
	Endometrial Volume (mm^3^)	4.52 ± 1.00	5.33 ± 0.76	**0.001**
	Adjusted Endometrial Volume	8.30 ± 2.52	10.76 ± 2.62	**0.001**
Embryo Transfer Day	Endometrial Thickness (mm)	9.06 ± 1.30	10.15 ± 1.35	**0.001**
	Endometrial Volume (mm^3^)	4.84 ± 1.01	5.59 ± 0.77	**0.001**
	Adjusted Endometrial Volume	8.32 ± 2.58	10.83 ± 2.73	**0.001**

Ratios in percentages (%) and mean values with standard deviation (SD). *rhCG* recombinant human chorionic gonadotropin

**Fig. 2:**
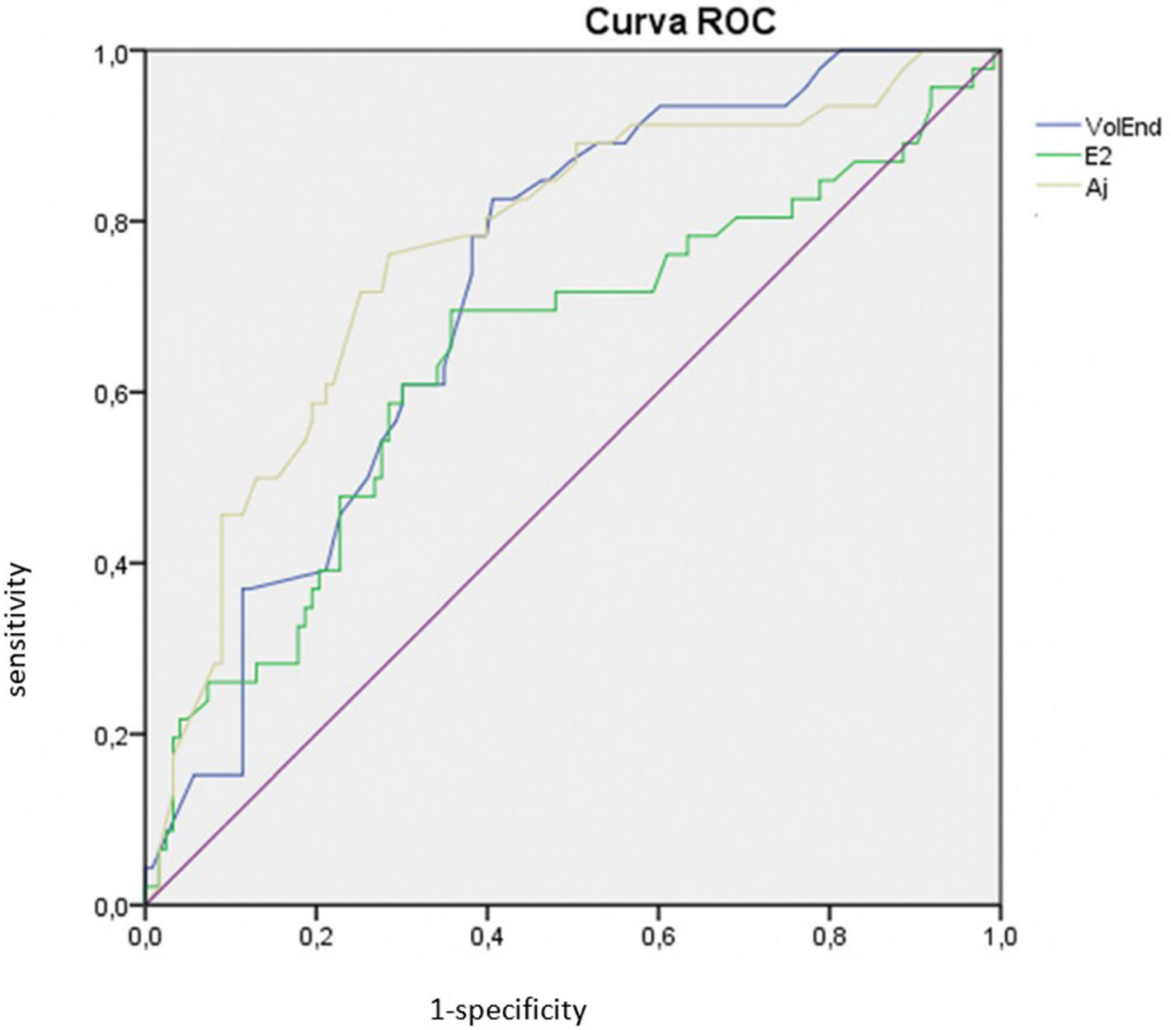
The ROC area under curves for prediction of positive outcome. Vol End – Endometrial Volume; E2 – Serum Estradiol; Aj – Adjusted Endometrial Volume

Receiver operating characteristic (ROC) curve analysis and comparison of area under curves (AUC) were performed for Estradiol, Endometrial Volume and Adjusted Endometrial Volume on the prediction of positive outcome with sensitivity (true positive rate, y-axis) and 1-specificity (false positive rate, x-axis). Values of 0,701; 0,723 and 0,756 were obtained respectively for the examined parameters. (Fig. 2).

In this study the intra-observer reliability was 0.96. In addition, because all measurements were performed by the same operator in this study there was no inter-observer variability.

## Discussion

The process of endometrial transformation from proliferative phase to secretory phase under the steroids hormonal influence, called endometrial decidualization is a set goal for optimal implantation. Cyclic changes of endometrium are regulated by ovarian hormones [18]. This pattern may alter due to the supraphysiological hormonal levels during ART cycles.

Single analysis of endometrial pattern at trigger day has been the most used, with contradictory findings. Recent studies (*Silva Martins, R.* et al.) have proven that perhaps serial evaluations provide better understanding rather than a single scoop at a pre-determined phase of the process [17, 19]. The main purpose of this study was to assess both parameters not only in a single scope pre-determined moment but with a serial prospective continuous evaluation.

Conflicting results have been published, but this new methodological approach to endometrial assessment during ART procedures, may shine a new light in better understating the way endometrium transforms and becomes receptive for successful embryo implantation. Serial evaluations, of both biochemical and biophysical parameters, better translate endometrium transformations and may be a base for the understating of endometrial receptivity [20, 21].

Endometrial volume and adjusted endometrial volume proven to be more effective with differences shown since the beginning of ovarian controlled stimulation. Both groups were similar at baseline but as soon as controlled ovarian stimulation started, the differences between those with a positive outcome and those without were clearly met.

In a similar way the rise of serum estradiol was significantly higher in the positive outcome group, despite the fact that the number of oocytes on pick up, mature oocytes and number of cleaved and blastocysts was similar between both groups. This reflects the effects that serum estradiol has directly on endometrium in later phases of its development prior to embryo implantation.

Serum estradiol and endometrial volume, may prove to be a useful management tool for clinicians in order to establish yet another diagnostic tool for better decision making in selective embryo transfer. This new methodology uptake, and a new perspective of endometrial analysis is certainly the strongest factor of this study, as well as the number of continuous serial evaluations on the same patient, throughout the ovarian controlled stimulation and its effects on endometrium. Still in our work, in line with previous publications positive outcomes in term of clinical pregnancies were met for values of serum estradiol at trigger day bellow 3000 pg/mL. This can be explained by the policy of tailor made controlled ovarian stimulation protocol, in mild stimulation to avoid potential OHSS.

We could not refrain to uphold expectation of these results as they show a serial of values, demonstrating a certain pattern of evolution on a transforming living tissue and its natural adaptations to a complex and yet unknown process. Also, the number of subjects in this study constitutes a limitation and further larger studies must be carried out in order to certainly establish this promising results.

## Conclusions

The underlying mechanism that results in failure of implantation of a good quality embryo on a supposed receptive endometrium is still unclear. Endometrial receptivity is still up to this date a controversial subject and a hot topic for discussion and analysis.

Most published studies present conflicting results and are still controversial. The adaptative changes and continuous evolution of the endometrium makes it difficult to establish a normative pattern of development in way to provide useful information regarding its receptiveness, in real time and in a personalized individualized setting.

This study showed that endometrial volumetry may identify a receptive endometrium as soon as day 6 of ovarian controlled stimulation. Serum Estradiol also showed some predicting value, with statistical significance. Nevertheless, the difference noted between groups did not affect the number of oocytes in oocyte retrieval, nor the number of mature oocytes. Also, the number of cleaved embryos and blastocysts was similar in both groups. This shows that serum estadiol has potential effects on endometrium development in later phase of its development. In this way clinicians may be made aware of this possibility and further enhance its procedures with better knowledge weather or not to perform embryo transfer on that given cycle.

## Data Availability

Encrypted non-disclosure data available at Open Science Framework database for peer review purpose only. Project name Physical Biomarkers in Endometrial Receptivity with access link: https://osf.io/hr25m/?view_only=8d5f6dcb8b25420bbd9188382163e7d7

## Abbreviations

ART: Assisted reproductive technology
CHCB: Centro Hospitalar Cova da Beira
ER: Endometrial receptivity
ERA: Endometrial receptivity array
GCP: Good clinical practices
hCG: human chorionic gonadotropin
ICH: International Conference Harmonisation
IU: International Units
SD: Standard deviation
WOI: Window of implantation
2D: Two dimensions
3D: Three dimensions
